# Supplementary figures and images for: Condensin positioning at telomeres by shelterin proteins drives sister-telomere disjunction in anaphase
Source: eLife. 2023 Nov 21;12:RP89812. doi: 10.7554/eLife.89812 (PMC10662949; doi:10.7554/eLife.89812)

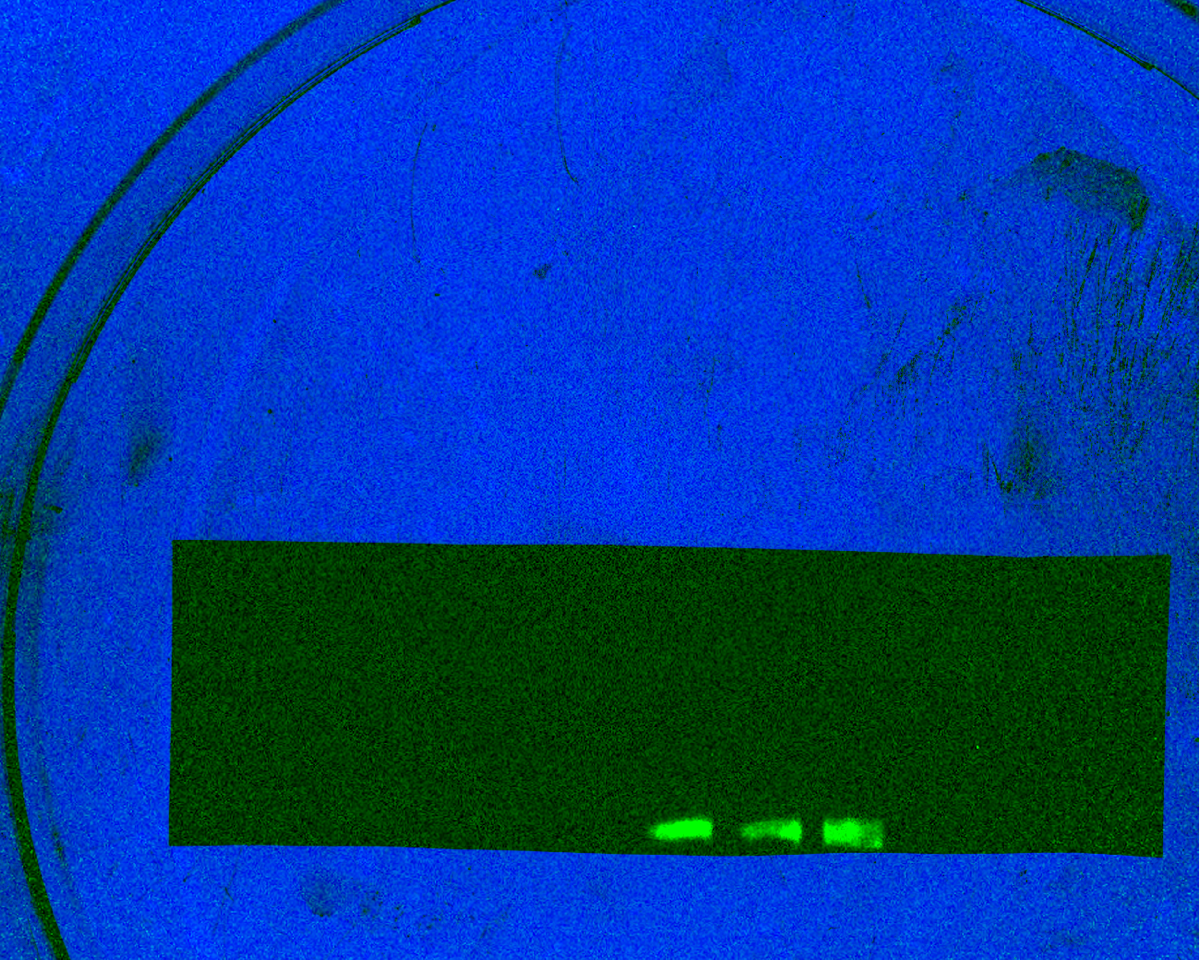

Supplement: Figure 1—source data 1. — Raw data of the western blot shown in Figure 1E. [file elife-89812-fig1-data1.zip › Bernard 2023-04-11 16h43m58s(1) (Multichannel).tiff]

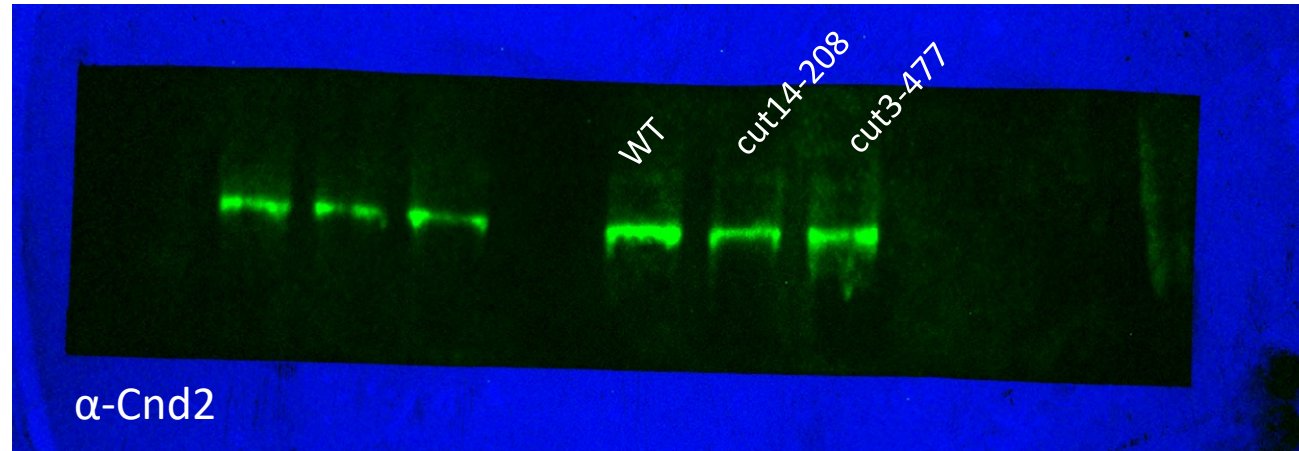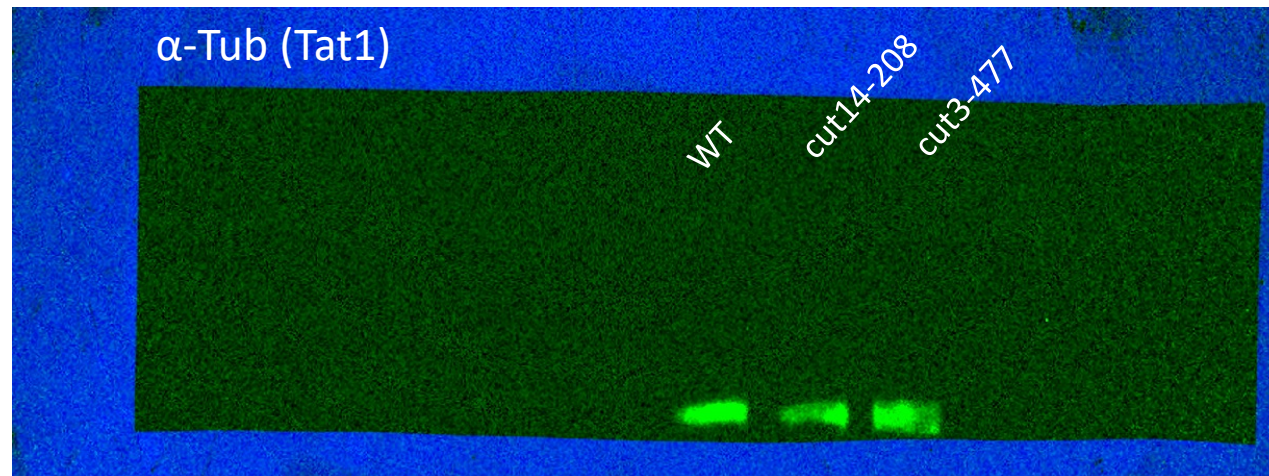

Supplement: Figure 1—source data 1. — Raw data of the western blot shown in Figure 1E. [file elife-89812-fig1-data1.zip › rawdata_Figure 1E.pdf]

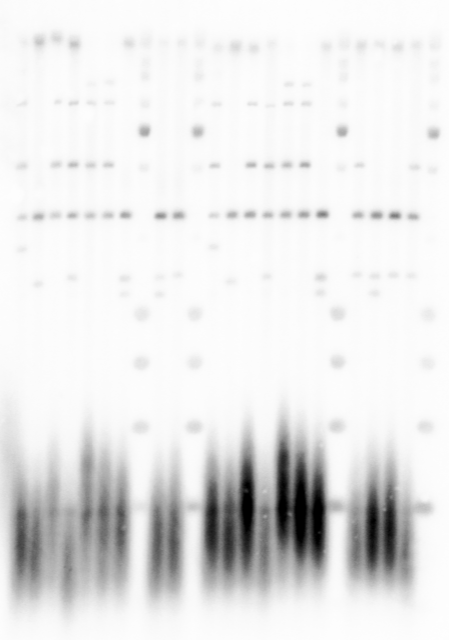

Supplement: Figure 2—source data 1. — The first part of the gel from the left has been cut out for Figure 2C. [file elife-89812-fig2-data1.zip › blotFig2 (1).tiff]

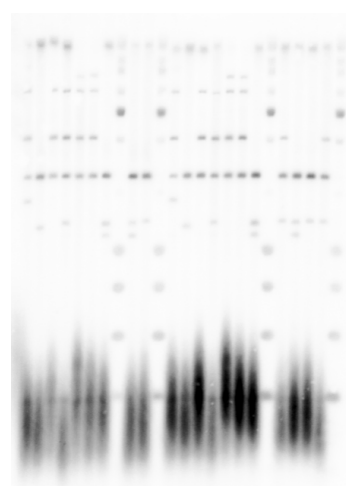

Supplement: Figure 2—source data 1. — The first part of the gel from the left has been cut out for Figure 2C. [file elife-89812-fig2-data1.zip › Fig 2 blot.pdf]
