# Supplementary material for: Condensin positioning at telomeres by shelterin proteins drives sister-telomere disjunction in anaphase
Source: eLife. 2023 Nov 21;12:RP89812. doi: 10.7554/eLife.89812 (PMC10662949; doi:10.7554/eLife.89812)
Supplement: Supplementary file 1. — The strain number, genotype, and figures corresponding to the use of these strains are indicated. [file elife-89812-supp1.docx]

| Strain | Genotype | Figures |
| --- | --- | --- |
| LY4682 | *h- leu1-32 ura4D18 cdc2asM17* | Fig. 1B |
| LY4731 | *h- leu1-32 ura4D cdc2asM17 cnd2-GFP-LEU2* | Fig. 1B |
| LY6281 | *Mata ade2-1 his3-11 his3-15 ura3 leu2-3 trp1-1 can1-100 SMC3-GFP-KanR* | Fig. 1C-D, S1, 4 & S4 |
| LY4483 | *h- leu1-32 ura4D-18 ura4-Pnmt41-slp1+ cnd2-GFP-LEU2* | Fig. 1C-E, S1, 3 & S3, 4 & S4 |
| LY6305 | *h- leu1-32 ura4D cut14-208 ura4-Pnmt41-slp1+ cnd2-GFP-LEU2* | Fig. 1C-E, S1D, 3 & S3, S4B |
| LY6304 | *h+ leu1-32 ura4D cut3-477 ura4-Pnmt41-slp1+ cnd2-GFP-LEU2* | Fig. 1E, S1E |
| LY5480 | *leu1- ura4- ade6- ura4-Pnmt41-slp1+ aur1::padh21-NLS-GFP-9PK::aur1R* | Fig. 4A, 4C, S4A & S4D |
| LY5298 | *h? leu1-32 ura4D18 ade6-21? KanR-Pnmt1-slp1+ taz1∆::ura4+ cnd2-GFP-LEU2* | Fig. 4A, 4C, S4A |
| LY5948 | *h- leu1-32 ura4D ura4-Pnmt41-slp1+ rap1∆::KanR cnd2-GFP-LEU2* | Fig. 4A, 4C, S4A |
| LY5898 | *h+ leu1-32 ura4D ura4-Pnmt41-slp1+ mit1∆::KanR cnd2-GFP-LEU2* | Fig. 4D-E, S4D-E |
| ST1711  ST2624  ST1738  ST2622  ST1665  FY24566  ST2640 | *h+ cdc11-CFP::KanR taz1-GFP::KanR mis6-2mRFP::hph*  *h?cut3-477 cdc11-CFP::KanR taz1-GFP::KanR mis6-2mRFP::hph*  *h? cut14-208 cdc11-CFP::KanR taz1-GFP::KanR mis6-2mRFP::hph*  *h?cut3-477 cdc11-CFP::KanR taz1-GFP::KanR*  *h?* *top2-250 cdc11-CFP:: KanR taz1-GFP:: KanR*  *h+ imr3L-tetO-ura4+ Z:adh31-tetR-tdTomato<<natr his7+<<Pdis1-GFP-lacI leu1 ade6 tel1L-lacO-kanr C:adh21-gar1-CFP<<hygr* (Tada et al. 2011)  *h? cut14-208 imr3L-tetO-ura4+ Z:adh31-tetR-tdTomato<<natr his7+<<Pdis1-GFP-lacI leu1 ade6 tel1L-lacO-kanr C:adh21-gar1-CFP<<hygr* | Fig. 2A-B  Fig. 2B  Fig2A & Fig.S2A  Fig.2D, 5C, 6A  Fig.2D-E  Fig.S2B  Fig.S2B |
| PR109 | *h- leu1-32 ura4D-18* | Fig. 5B, S2C |
| SC388 | *h- leu1-32 ura4D-18 ade6-M210 taz1 ::ura4+* | Fig. 5B |
| LY999 | *h- leu1-32 ura4D ade6-M210 cut3-477 :NatR* | Fig. 5B, S2C |
| SC1432 | *h ? leu1-32 ura4D-18 taz1::ura4+ cut3-477 :NatR* | Fig. 5B |
| ST1928  ST1664  ST1172  ST1183  ST1657  ST2636  LY646 | *h? taz1::ura+ cut3-477 ccq1-GFP::KanR cdc11-CFP:: KanR ura4D-18*  h? *top2-250 ndc80-GFP:: KanR cdc11-CFP:: KanR mis6-RFP-hyg*  *h? ccq1-GFP:: KanR cdc11-CFP:: KanR*  *h- taz1::KanR ccq1-GFP::KanR cdc11-CFP:: KanR*  *h- cut3-477 ccq1-GFP:: KanR cdc11-CFP :: KanR*  *h? mit1::hph cdc11-CFP:: KanR taz1-GFP:: KanR*  *h- leu1-32 ura4D-18 cut14-90* | Fig. 5A  Fig. S2C  Fig. 5A  Fig. 5A  Fig. 5A  Fig. 5C  Fig. 2C |
| LY140 | *h- leu1-32 ura4D cut14-208* | Fig. 2C |
| LY1252 | *h- leu1-32 ura4D ade6-M210 cut14-180* | Fig. 2C |
| LY3185 | *h- ura4D ade6-M210 cut3-M26* | Fig. 2C |
| LY3186  LY4681  LY7260  LY7262  STCS138  ST2638 | *h- ura4D ade6-M210 cut3-I23-GFP:his7+*  *h+ leu1-32 ura4D18 cdc2asM17*  *h+ leu1-32 ura4D? ade6-210 cdc2asM17 psm3-GFP-NatR*  *h+ leu1-32 ura4D? ade6-210 cdc2asM17 psm3-GFP-NatR cut3-477*  *h? rad21-K1 cdc11-CFP:: KanR taz1-GFP:: KanR*  *h? rad21-K1 cut3-477 cdc11-CFP:: KanR taz1-GFP:: KanR* | Fig. 2C  Fig. 6B, S5  Fig. 6B, S5  Fig. 6B, S5  Fig.6A, S6A  Fig.6A, S6A |
|  |  |  |
|  |  |  |
