# Supplementary material for: Condensin positioning at telomeres by shelterin proteins drives sister-telomere disjunction in anaphase
Source: eLife. 2023 Nov 21;12:RP89812. doi: 10.7554/eLife.89812 (PMC10662949; doi:10.7554/eLife.89812)
Supplement: Supplementary file 2. — The forward and reverse primers used in this study are indicated. [file elife-89812-supp3.docx]

| Site | Forward primer | Reverse primer |
| --- | --- | --- |
| cnt1 | accgttgcaacttacatcagc | ggtcgccaaatagcaatgag |
| exg1 | cacatagacggaccactttgag | atatgtcacctgtggctgagtg |
| gas1 | AATAGCATGTCGAGGTTGTATGG | TGTCATCGCGAAACCTTACC |
| cnd1 | agcaattagccgaacgtctg | caccacatgatcccattgac |
| cdc22 | CGGGCTAAATTGAGGTATGG | CGCAGTTGCACTTTTCAAAC |
| gly05 | gacgttgtgctaaaaggtgttg | ggaaatcgagcagaggtcag |
| rRNA37 | taggatcgctgagaatccatc | tggattaaaacacattgcttgc |
| arg04 | cattaatccgccgtggatag | ttcacctaatagttgccaaacg |
| cendh1 | cgctttgttgtcgtggacta | aacacggcgataagaaatgg |
| kgd1 | GCTTCAGATCATTTGGTCCAG | GGAATTCATGGCATTGGAAC |
| lvs1 | GGCATTTGTCGGTAACACTC | GTTTGCAGCGACTGTGTTTC |
| tel30 | GAAATTGTGCCACGTTGGAG | GACAGGGTCCTTGCTAAGTTTC |
| tel19 | CGGGATAACACACATGCAAC | GCTTTGATGGCAACTGGTTC |
| tel13 | ccaatccccaggtttctttc | tttcgacctatcagcggttg |
| tel9.2 | ACACGCTCTGACAACATTCG | CGCAATCTCGATTACCGAAC |
| tel6.1 | AAACAACTGCAAGCGGTAGG | CGCATTTACCATTCCTCCAC |
| tel2.4 | AGCAGGGGACTATATTGGAGTG | CCCCTTCAATTACCAAAGTCCAC |
| tel0 | GTGTGGAATTGAGTATGGTGAA | CGGCTGACGGGTGGGGCCCAATA |
| isl7 | ACACTTGTTTCAGCCGATTTC | AAGCATTGCTCCATTAAAACAAC |
| isl13 | ATGAAGGTACGGAAGCAACG | TAGCCCTTTCATTAATAGCTTCG |
| isl15 | AGAAAAGGCAATGCGAGAGC | CGCAAATCATCTGACATTGG |
| scCARIV | TCAGGGAAGGTACGGAAATG | GCATGACTATTCGCGTTTGAG |
| scCEN4 | AAATGCCGAGGCTTTCATAG | GTGACGATAAAACCGGAAGG |
