# Supplementary material for: Condensin positioning at telomeres by shelterin proteins drives sister-telomere disjunction in anaphase
Source: eLife. 2023 Nov 21;12:RP89812. doi: 10.7554/eLife.89812 (PMC10662949; doi:10.7554/eLife.89812)
Supplement: Supplementary file 3. [file elife-89812-supp6.docx]

| Antibody | target | experiment |
| --- | --- | --- |
| A-11122 (Invitrogen) | Anti-GFP | ChIP |
| Tat-1 (a gift from Keith Gull) | Anti-α-Tubulin | Western blot |
| Cnd2 (euromedex, GTX64102) | Anti-Cnd2 fission yeast | Western blot |
| anti-rabbit NA9340V Amersham | Anti-rabbit secondary | Western blot |
| anti-mouse NA931 -1ml Amersham | Anti-mouse secondary | Western blot |
